# Dynamic and Static Behavior of Hollow-Core FRP-Concrete-Steel and Reinforced Concrete Bridge Columns under Vehicle Collision

**DOI:** 10.3390/polym8120432

**Published:** 2016-12-13

**Authors:** Omar I. Abdelkarim, Mohamed A. ElGawady

**Affiliations:** 1 Civil Engineering Department, University of Sherbrooke, Sherbrooke, QC J1K 2R1, Canada; Abdelkarim.Omar@Usherbrooke.ca; 2 Department of Civil, Architectural, and Environmental Engineering, Missouri University of Science and Technology, Rolla, MO 65409, USA

**Keywords:** HC-FCS column, RC column, composite column, impact loading, LS-DYNA

## Abstract

This paper presents the difference in behavior between hollow-core fiber reinforced polymer-concrete-steel (HC-FCS) columns and conventional reinforced concrete (RC) columns under vehicle collision in terms of dynamic and static forces. The HC-FCS column consisted of an outer FRP tube, an inner steel tube, and a concrete shell sandwiched between the two tubes. The steel tube was hollow inside and embedded into the concrete footing with a length of 1.5 times the tube diameter while the FRP tube stopped at the top of footing. The RC column had a solid cross-section. The study was conducted through extensive finite element impact analyses using LS-DYNA software. Nine parameters were studied including the concrete material model, unconfined concrete compressive strength, material strain rate, column height-to-diameter ratio, column diameter, column top boundary condition, axial load level, vehicle velocity, and vehicle mass. Generally, the HC-FCS columns had lower dynamic forces and higher static forces than the RC columns when changing the values of the different parameters. During vehicle collision with either the RC or the HC-FCS columns, the imposed dynamic forces and their equivalent static forces were affected mainly by the vehicle velocity and vehicle mass.

## 1. Introduction

Bridges are under the risk of vehicular impact, especially due to recent increases in traffic flow. In the United States, surveys reported that, between 1980 and 2012, approximately 15% of bridge failures occurred because of vehicular impact. This represented the third-highest cause of bridge failures [1]. Moreover, the studies showed that the number of reported bridge collisions are linked to increased construction activity [2]. For example, it was found that the number of annual bridge impacts increased from 69 to 219 in New York State during the period of 2000–2005 due to the growth of real estate during this period [2].

Different studies have been conducted on the behavior of reinforced concrete (RC) columns under vehicle collision [3,4,5,6]. The American Association of State Highway and Transportation Officials-Load and Resistance Factor Design (AASHTO-LRFD) Bridge Design Specifications [7] require that the unshielded pier or abutment be designed for a constant equivalent static force of 2670 kN (600 kips), which is assumed to act in a direction of zero to 15 degrees with the edge of the pavement in a horizontal plane, at a distance of 1.5 m (5.0 ft) above ground. Abdelkarim and ElGawady [3] concluded that the design force of the AASHTO-LRFD is quite conservative in some cases and nonconservative in others, the reason being that the AASHTO-LRFD uses a constant design force regardless of truck characteristics. Also, they presented the first equation to design RC columns under vehicular impact using the vehicle mass and velocity (Equations (1) and (2) for International System (SI) and custom units, respectively). Using this approach, bridge designers could design different bridge columns on different highways based on the predicted truck loads and speeds documented in the highway survey.

(1) $$KEB_{ESF}=33\sqrt{m\;v_{r}^{2}}=46\sqrt{KE}$$

where *KEB_ESF_* = kinetic energy-based equivalent static force (kN), *m* = the vehicle mass in tons, *v_r_* = the vehicle velocity in m/s, and *KE* = $\frac{1}{2}\;mv_{r}^{2}$ = kinetic energy of the vehicle in kN.m.

(2) $$KEB_{ESF}=2.1\sqrt{m\;v_{r}^{2}}=3\sqrt{KE}$$

where *KEB_ESF_* = kinetic energy-based equivalent static force (kip), *m* = the vehicle mass in kip, *v_r_* = the vehicle velocity in mph, and *KE* = $\frac{1}{2}\;mv_{r}^{2}$ = kinetic energy of the vehicle in kip.mph^2^.

Most of the RC columns are constructed with solid cross-sections. Nonetheless, hollow-core cross-sections are preferable for very tall bridge columns to reduce the mass and to reduce freight costs for precast columns. However, although the hollow-core RC column with one layer of longitudinal reinforcement is easy to construct, it showed brittle behavior under seismic loading [8,9]. Therefore, two coaxial longitudinal reinforcements connected by extensive cross ties and transversal reinforcement should be used to reach good ductility for the hollow-core RC columns [10,11,12]. Recently, Teng et al. [13] introduced a new hollow-core column consisting of an outer fiber-reinforced polymer (FRP) tube, an inner steel tube, and a concrete shell sandwiched between them. This new type was called hollow-core FRP-concrete-steel (HC-FCS). The steel tube is hollow inside, and the FRP tube stops at the top of the footing for full utilization of its confinement of the concrete shell. The steel tube works as longitudinal and shear reinforcement. There are no other reinforcements used in the column. The existence of the FRP tube in the outer surface of the concrete shell and the steel tube in the inner surface eliminates the concrete shrinkage as it significantly reduces water evaporation. Both tubes provide continuous confinement of the concrete shell, achieving high ductility. The HC-FCS column uses 60%–75% less concrete material than the solid cross-sectional RC column. Such a column is easy to construct as the two tubes act as formworks for the concrete shell. Abdelkarim et al. [14] reported that the construction of the HC-FCS columns took approximately 10% of the construction time of the RC column with solid cross-section at the High-Bay Structures Lab at the Missouri University of Science and Technology.

HC-FCS columns have been investigated extensively under axial, flexural, and combined axial-flexural loadings [15,16,17,18,19,20]. The studies showed that the HC-FCS columns exhibited high ductility under different loads. Under the flexural loading, the maximum lateral drift of the HC-FCS columns is controlled by the FRP confinement while the steel tube controls the flexural strength [15]. Abdelkarim and ElGawady [21] investigated the performance of the HC-FCS columns under vehicular impact. They found that the main resistance of such columns to vehicular impact comes from the steel tube only. Also, they presented the first equation to design the HC-FCS columns under vehicular impact (Equations (3) and (4) for SI and custom units, respectively).

(3) $$KEB_{ESF}=42\sqrt{m\;v_{r}^{2}}=60\sqrt{KE}$$

where *KEB_ESF_* = kinetic energy-based equivalent static force (kN), *m* = the vehicle mass in tons, *v_r_* = the vehicle velocity in m/s, and *KE* = $\frac{1}{2}\;mv_{r}^{2}$ = kinetic energy of the vehicle in kN.m.

(4) $$KEB_{ESF}=2.7\sqrt{m\;v_{r}^{2}}=3.9\sqrt{KE}$$

where *KEB_ESF_* = kinetic energy-based equivalent static force (kip), *m* = the vehicle mass in kip, *v_r_* = the vehicle velocity in mph, and *KE* = $\frac{1}{2}\;mv_{r}^{2}$ = kinetic energy of the vehicle in kip.mph^2^.

The concrete shell of the HC-FCS column is confined from outside by the FRP tube through confinement pressure ($f_{l}$). The $f_{l}$ is the FRP lateral pressure induced when the concrete starts to expand under the axial compressive stress. The confinement pressure and the confinement ratio could be calculated using Equations (5) and (6):

(5) $$Confinement\;pressure\;\left(f_{l}\right)=\frac{2\;E_{f}\;\varepsilon_{f}\;t_{f}}{D}$$

(6) $$Confinement\;ratio\;\left(CR\right)=\frac{f_{l}}{f_{c}^{\prime }}$$

where $E_{f}$ is the elastic modulus of the FRP tube in the confinement direction, $\varepsilon_{f}$ is the ultimate tensile strain of the FRP in the confinement direction, $t_{f}$ is the FRP tube thickness, D is the column’s diameter, and $f_{c}^{\prime }$ is the characterized unconfined concrete cylindrical strength.

This paper presents a comparison between the RC and HC-FCS columns in terms of dynamic and static forces under vehicular impact through finite element (FE) parametric study. The dynamic force is represented by the peak dynamic force (PDF) during the event of a vehicle collision with a bridge column. The equivalent static force is represented by the peak of the 25 millisecond moving average (PTMSA) of the time-dynamic force relation curve when a vehicle collides with a bridge column [22]. The PTMSA was used by Abdelkarim and ElGawady [3,21] for developing their Equations (1)–(4) as the equivalent static force to design the bridge columns under vehicular impact. LS-DYNA [23] software was used to conduct the study.

## 2. Parametric Study

The validation of the FE models of the HC-FCS and RC columns using LS-DYNA software (Livermore Software Technology Corporation (LSTC), Livermore, CA, USA) was explained by Abdelkarim and ElGawady [17,22], respectively. Each type of column was hit by an errant truck, and the dynamic and equivalent static forces of the collision events were recorded for comparison. Both columns were designed to have almost the same axial compressive load capacity (*P_o_*) which can be calculated using Equation (7) [24]. The confined concrete strength ($f_{cc}^{\prime })$ of the HC-FCS column’s concrete shell was considered instead of the $f_{c}^{\prime }$ in calculating the column’s axial capacity. The $f_{cc}^{\prime }$ was determined using Yu et al.’s [25] confinement model. The distance between the errant vehicle and the column was 150 mm (0.5 ft). The soil depth above the footing for each type of column was 1000 mm (3.3 ft). The comparison between the RC and HC-FCS columns under vehicle collision was conducted on the dynamic force (PDF) and equivalent static force (PTMSA) through nine parameters as follows:

- Concrete material model (elastic and nonlinear)
- Unconfined concrete compressive strength ($f_{c}^{\prime })$ ranging from 20.7 MPa (3000 psi) to 69.0 MPa (10,000 psi)
- Material strain rate (SR, both considered and not considered)
- Column height-to-diameter ratio (*H/D_o_*) ranging from 2.5 to 10.0
- Column diameter (*D_o_*) ranging from 1200 mm (4.0 ft) to 2100 mm (7.0 ft)
- Column top boundary condition (free, superstructure, and hinged)
- Axial load level (*P/P_o_*) ranging from 0% to 10%
- Vehicle velocity (*v_r_*) ranging from 32 kph (20 mph) to 112 kph (70 mph)
- Vehicle mass (*m*) ranging from 2 tons (4.4 kips) to 30 tons (65 kips)

Twenty-one variables were examined for each type of columns with a total of 42 investigated columns in this study. The range of selected variables regarding the examined parameters of the investigated columns is summarized in Table 1. Columns C0-R and C0-H were used as the reference columns for the RC and HC-FCS columns, respectively. It should be noted that some of the selected parameters may be uncommon in practice. However, they were used to fully understand the columns’ performance under a wide spectrum of parameters. One parameter was investigated in each group, and the rest were kept constant as in the reference column. For example, when the column diameter changed, the axial load value changed correspondingly in order to keep the axial load level of *P_o_* constant.

## 3. Finite Element Analyses

### 3.1. Geometry of the Investigated Columns

**Figure 1:**
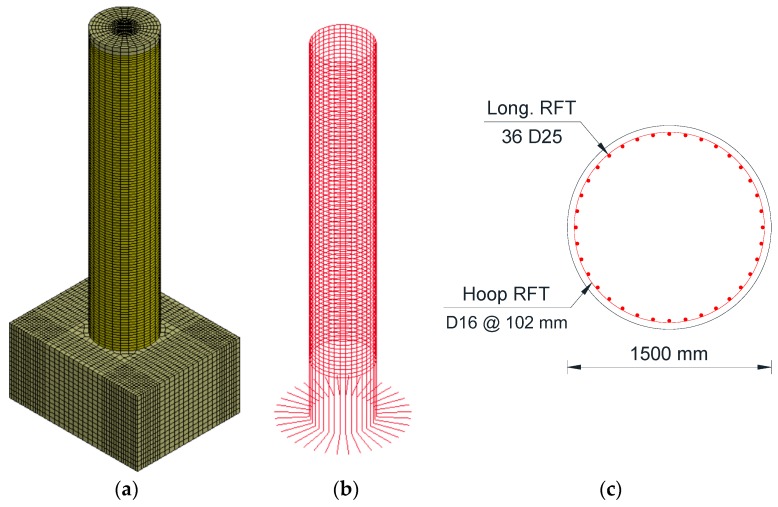
Modeling of the C0-R column: (**a**) overall view of the column and (**b**) longitudinal and hoop reinforcements; and (**c**) cross-section.

All of the modeled RC columns have solid cross-sections with longitudinal reinforcement ratios of 1.0% of the cross-sectional gross area. The transversal hoop reinforcement used was D16 @ 102 mm (#5 @ 4.0 inches) for all of the columns. An axial load (*P*) was applied on the column representing a percentage of the *P_o_* as in Table 1. The *P_o_* was calculated using Equation (7) [24]. Figure 1 illustrates the “C0-R” reference column components. The C0-R column had a diameter of 1500 mm (5 ft) and a height of 7620 mm (25 ft) with a height-to-diameter ratio (*H/D_o_*) of 5.0. The column had longitudinal reinforcements of 36 D25 (#8) and hoop reinforcements of D16 @ 102 mm (#5 @ 4.0 inches). The $f_{c}^{\prime }$ of the column was 34.5 MPa (5000 psi).

(7) $$P_{o}=\;A_{s}f_{y}+0.85\;f_{c}(A_{c}-A_{s})$$

where *P_o_* = the axial compressive load capacity of the column, $A_{s}$ = the cross-sectional area of the longitudinal reinforcement or steel tube, $A_{c}$ = the concrete cross-sectional area of the column, $f_{y}$ = the yield stress of the longitudinal reinforcement or steel tube, and $f_{c}$ = the cylindrical concrete unconfined compressive strength $\left(f_{c}^{\prime }\right)$ for the RC column or the cylindrical concrete confined compressive stress $\left(f_{cc}^{\prime }\right)$ for the HC-FCS column.

**Figure 2:**
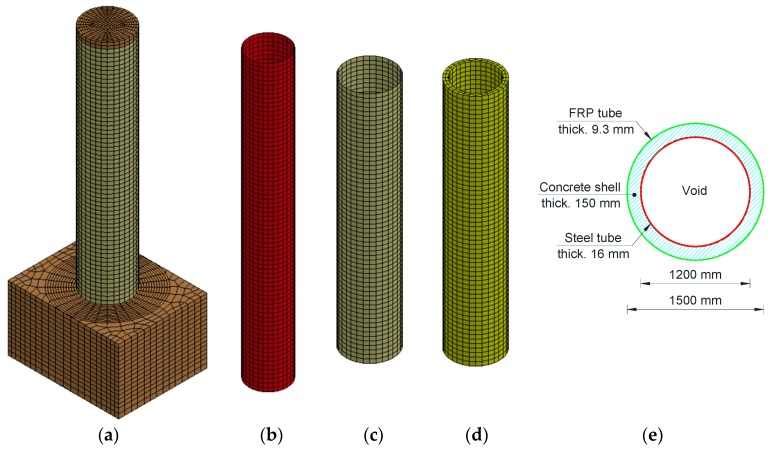
Modeling of the C0-H column: (**a**) overall view of the column; (**b**) steel tube; (**c**) FRP tube; (**d**) concrete shell; and (**e**) cross-section.

All of the HC-FCS columns consisted of an outer Glass FRP tube, an inner steel tube, and a concrete shell sandwiched between the two tubes. The FRP tube stopped at the top of the footing while the steel tube was embedded into the footing with a length (*L_e_*) of 1.5 times the outer diameter of the steel tube (*D_i_*). The steel tube was hollow inside. The *D_i_* changed with the change of the column’s outer diameter (*D_o_*) achieving same void ratio (*D_i_*/*D_o_*) of 0.80. The diameter-to-thickness (*D_i_*/*t_s_*) of the steel tube was taken as 75 for all of the HC-FCS columns. Hence, the steel tube thickness (*t_s_*) changed with the change of the *D_i_*. The thickness of the FRP tube (*t_f_*) varied with changing the *D_o_* and $f_{c}^{\prime }$ to keep a constant confinement ratio of 0.10. Figure 2 illustrates the “C0-H” reference column components. The C0-H column had *D_o_* of 1500 mm (5.0 ft) and *D_i_* of 1200 (47.2 inch) with a thickness of 16.0 mm (0.63 inch) and *D_i_/t_s_* of 75. The *Le* was 1.5 *D_i_* which equals 1800 mm (70.9 inch). The thickness of the outer FRP tube was 9.3 mm (0.37 inch) with a confinement ratio of 0.1. The column’s height was 7620 mm (25.0 ft) with a height-to-diameter ratio (*H/D_o_*) of 5.0. The $f_{c}^{\prime }$ was 34.5 MPa (5000 psi).

All of the RC and HC-FCS columns were hinged at the top boundary condition except columns C11-R, C11-H, C12-R, and C12-H. Columns C11-R and C11-H had a top free boundary condition. The same superstructure was applied on the top of columns C12-R and C12-H instead of the hinged condition. The superstructure utilized was developed by El-Tawil et al. [6] for an existing bridge in Florida which consisted of two adjacent steel girders which was comprised of a composite steel-concrete box girder with two unequal spans of 53,340 mm (175 ft) and 50,290 mm (165 ft). Pinned supports were applied at the far ends of the girders while the bridge bearings were used below the superstructure at the column location. Elform_2 element type was used to simulate the steel girders with a cross-sectional area of 80,000 mm^2^ (124 inch^2^). The moment of inertia in the strong direction (*I*_yy_ about the vertical axis) was 8.3 × 10^10^ mm^4^ (2.0 × 10^5^ inches^4^) and the moment of inertia in the weak direction (*I*_zz_ about the horizontal axis) was 2.8 × 10^10^ mm^4^ (6.7 × 10^4^ inches^4^). Also, Elform_2 was used to simulate the bearings which were 37 mm (1.5 inches) thick and 200 mm × 200 mm (8 × 8 inches) in the cross-section with shear modulus of 0.61 MPa (88.0 psi).

### 3.2. FE Modeling

For the RC columns, the concrete column and footing were modeled using one-point quadrature solid elements with hourglass control (Control_Hourglass). Such elements use constant stress through the element while the local deformation is to be assigned using the hourglass modes. The hourglass control is defined as the zero-energy deformation modes associated with the one-point-quadrature element resulting in a non-constant strain field in the element to imitate the full integration elements but with a much shorter solution time. The value of hourglass (Hourglass_QH) was taken as the default value of 0.1. The hourglass type (Hourglass_IHQ) was taken as type 4 (stiffness form_Flanagan-Belytschko). The detailed information on the hourglass equations are described in the LS-DYNA theory manual [23]. The concrete cover of the column was assigned to spall using the “add-erosion” for concrete material when the axial strain exceeds the value of 0.005 [26] to represent the reality of when a vehicle hits a column. The longitudinal and hoop reinforcements were modeled using truss_beam elements by defining the cross-sectional area of the reinforced rebar. For the HC-FCS columns, the concrete shell and footing were modeled using solid elements similar to that of the RC columns. The FRP and steel tubes were modeled using shell elements. In order to avoid excessive local damage of the columns’ top due to the applied axial load, a rigid cylinder with a diameter the same as the column diameter and a thickness of 200 mm (7.9 inches) was modeled using solid elements and placed atop all of the investigated columns. The coincident nodes at the column’s top surface and the rigid cylinder’s bottom surface were merged.

### 3.3. Interfaces among the Columns’ Components

For the RC columns, the beam elements of the hoop and longitudinal reinforcements were constrained to the concrete solid elements by ‘Lagrange-in-solid’, simulating the perfect bond of the reinforcements with the concrete column and footing. For the HC-FCS columns, the steel and FRP tubes were in contact with the concrete shell and footing using surface-to-surface contact elements with a coefficient of friction of 0.5 based on the validation conducted by Abdelkarim and ElGawady [17]. This type of contact element considers that any slip or separation could occurr between the inner surface of the concrete shell or footing and the steel tube or between the outer surface of the concrete shell and the FRP tube. The same type of contact element with a coefficient of friction of 0.5 was used between the bottom of the concrete shell and the top of the footing. Node-to-surface contact elements were used between the bottom of the FRP tube and the top of the footing and between the bottom of the steel tube and the footing with a coefficient of friction of 0.5.

### 3.4. Material Models

#### 3.4.1. Concrete Material Models

Two material models were investigated during this study; nonlinear model (mat72RIII) and elastic model (mat001). The nonlinear concrete model was used as the main model in the study as the AASHTO-LRFD considers vehicle impact as a type of extreme loading, and the nonlinear behavior is allowed. The parameters of mat72RIII were automatically generated using the $f_{c}^{\prime }$. All the generated parameters were taken as the default values. The strain rate effect was considered for all of the columns except the C5-R and C5-H columns. The effect of the strain rate is considered using the dynamic increase factor (DIF). The curve of strain rate-DIF relation was defined in the material mat72RIII according to the Equations (8)–(15) [27,28]. Elastic material mat001 was used for columns C1-R and C1-H, while mat72RIII was used for the other columns. The DIF was considered in the elastic modulus (E) equation by the ACI-318 [24] (E = 4700 × $\sqrt{\mathrm{DIF}\times f_{c}^{\prime }}$) and the Poisson’s ratio was taken as 0.20 [29].

(8) $$DIF_{c}=\frac{f_{c}}{f_{cs}}=\;{\left(\frac{\dot{\varepsilon }}{\dot{\varepsilon_{sc}}}\right)}^{1.026\;\alpha_{s}}\;\mathrm{for}\;\dot{\varepsilon }\leq 30\;s^{-1}$$

(9) $$DIF_{c}=\frac{f_{c}}{f_{cs}}=\;\gamma_{s}\;{\left(\frac{\dot{\varepsilon }}{\dot{\varepsilon_{sc}}}\right)}^{0.33}\;\mathrm{for}\;\dot{\varepsilon }>30\;s^{-1}$$

(10) $$\alpha_{s}={\left(5+9\frac{f_{cs}}{f_{co}}\right)}^{-1}$$

(11) $$log\gamma_{s}=6.156\;\alpha_{s}-2$$

where

*DIF_c_* = compressive strength dynamic increase factor
$\dot{\varepsilon }$ = strain rate in the range of 30 × 10^−6^ to 300 s^−1^
$\dot{\varepsilon_{sc}}$ = static strain rate of 30 × 10^−6^ s^−1^,
$f_{c}$ = the dynamic compressive strength at $\dot{\varepsilon }$
$f_{cs}$ = the static compressive strength at $\dot{\varepsilon_{s}}$
$f_{co}$ = 10 MPa = 1450 psi

(12) $$DIF_{t}=\frac{f_{t}}{f_{ts}}=\;{\left(\frac{\dot{\varepsilon }}{\dot{\varepsilon_{st}}}\right)}^{\delta }\;\mathrm{for}\;\dot{\varepsilon }\;\leq 1\;s^{-1}$$

(13) $$DIF_{t}=\frac{f_{t}}{f_{ts}}=\;\beta \;{\left(\frac{\dot{\varepsilon }}{\dot{\varepsilon_{st}}}\right)}^{0.33}\;\mathrm{for}\;\dot{\varepsilon }\;>1\;s^{-1}$$

(14) $$\delta ={\left(1+8\frac{f_{cs}}{f_{co}}\right)}^{-1}$$

(15) log β=6 δ−2

where

*DIF_t_* = tensile strength dynamic increase factor
$f_{t}$ = the dynamic tensile strength at $\dot{\varepsilon }$
$f_{ts}$ = the static tensile strength at $\dot{\varepsilon_{s}}$
$\dot{\varepsilon }$ = strain rate in the range of 10^−6^ to 160 s^−1^
$\dot{\varepsilon_{st}}$ = static strain rate of 10^−6^ s^−1^

#### 3.4.2. Steel Material Model

Plastic_kinamatic mat003 was used for the steel reinforcements for the RC columns and for the steel tube for the HC-FCS columns. The material properties were the elastic modulus (E), the yield stress, Poisson’s ratio, the tangent modulus, and the ultimate plastic strain which were taken as 200 GPa (29,000 ksi); 420.0 MPa (60,900 psi); 0.30; 1102 MPa (160 ksi); and 0.12, respectively [26]. The strain rate effect was considered for the dynamic yield strength using Equation (16) [30] while the elastic modulus was kept constant [31].

(16) $$f_{yd}=\;1+{\left(\frac{\dot{\varepsilon }}{c}\right)}^{\frac{1}{p}}$$

where $f_{yd}=$ dynamic yield stress and *p* and *c* were taken as 5 and 40, respectively.

#### 3.4.3. FRP Material Model

Orthotropic material 054_enhanced composite damage was used to simulate the FRP tubes of the HC-FCS columns. The material properties were the axial compression elastic modulus, hoop elastic modulus, axial ultimate compressive strength, and hoop rupture strength which were taken as 4.7 GPa (677 ksi); 20.8 GPa (3020 ksi); 83.8 GPa (12,510 ksi); and 276.9 GPa (40,150 ksi), respectively. The FRP tube material properties were referenced to the manufacturer’s data sheet of a filament winding tube with a fiber angle of ±55° [32]. The strain rate effect was considered using Equation (17) where C_rate_ was taken 0.03 for all of the strength values, shear moduli, and transverse modulus; and was taken 0.0 for the in-plane Young’s modulus [33]. The $\dot{\varepsilon_{0}}$ is the reference strain rate which equals to 1.0.

(17) $$E=\;\frac{\left\{E_{\mathrm{RT}}\right\}}{\left\{E_{0}\right\}}=1+\;\left\{C_{\mathrm{rate}}\right\}\ln\frac{\left\{\dot{\bar{\varepsilon }}\right\}}{\dot{\varepsilon_{0}}}$$

### 3.5. Trucks FE

**Figure 3:**
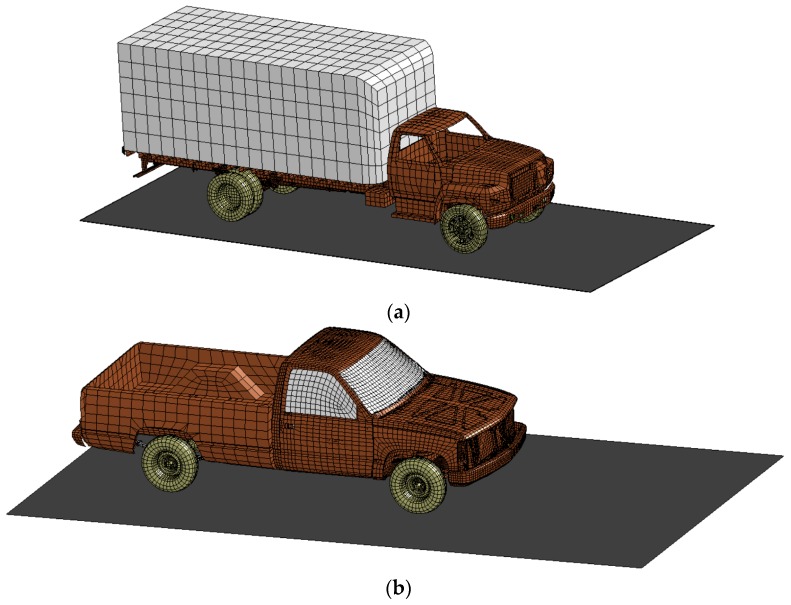
Modeling of the vehicles (NCAC [34]): (**a**) Ford Single Unit Truck (SUT) and (**b**) Chevrolet C2500 Pickup.

The detailed FE model (58,313 elements) Chevrolet C2500 Pickup [34] was used in the collision with columns C18-R and C18-H while the Ford Single Unit Truck (SUT) reduced FE model (35,353 elements) [34] was used in the collision with the other columns (Figure 3). These vehicles’ models were developed by the National Crash Analysis Center (NCAC) of George Washington University under a contract with both the Federal Highway Administration (FHWA) and the National Highway Traffic Safety Administration (NHTSA) of the U.S. Department of Transportation (DOT) and were downloaded from the NCAC website. The vehicle speed and mass were investigated during this research where the speed ranged from 32 kph (20 mph) to 112 kph (70 mph) while the mass ranged from 2 tons (4.4 kips) to 30 tons (65 kips). Automatic surface-to-surface contact elements by parts were used during the collision to make contact between the vehicle and the bridge columns. 

## 4. Results and Discussion

### 4.1. General Behavior

**Figure 4:**
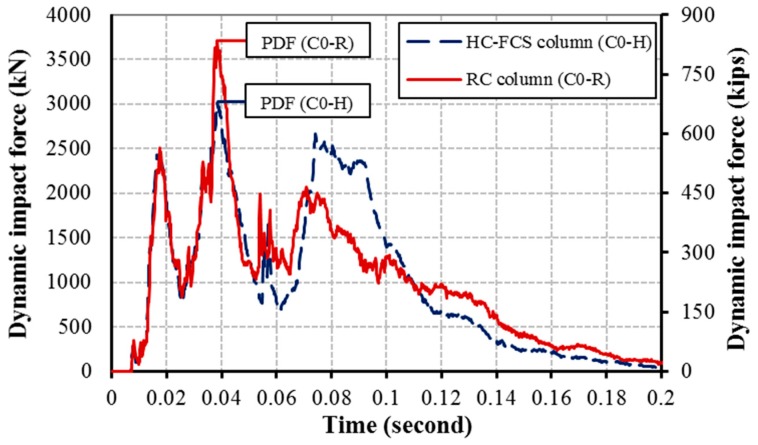
Time-dynamic impact force relationship of C0-R and C0-H columns.

**Figure 5:**
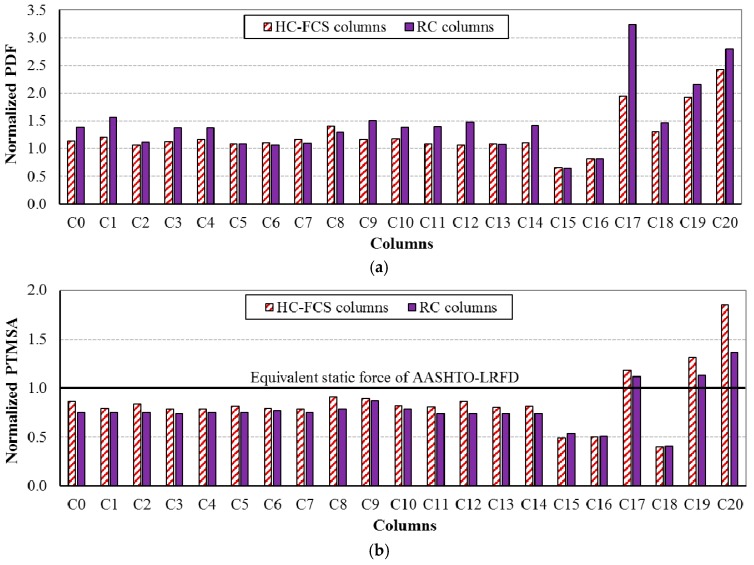
Normalized forces for all of the investigated columns: (**a**) normalized PDF and (**b**) normalized PTMSA.

Figure 4 illustrates the relation between the dynamic impact force and the event time during the vehicle collision with the columns C0-R and C0-H where the PDF of each column was pointed on the curve. The PTMSA, equivalent static force, of each column was calculated using the figure as the maximum value of the 25 millisecond moving average which was usually found around the time of the PDF. Figure 5 illustrates the normalized PDF and PTMSA for all of the investigated columns, where the normalized force was the calculated force divided by the equivalent static force from the AASHTO-LRFD specifications of 2670 kN (600 kips). In general, the HC-FCS columns had lower PDFs than the RC columns through changing the parameters, the reason being that the HC-FCS columns dissipated higher energy (in the form of steel tube deformation) than the RC columns, leading to lower PDF. This lower dynamic force would reduce the effect of the collision on the drivers during the event which may save human lives. However, in the other direction, the HC-FCS columns, generally, had higher PTMSA than the RC columns through changing the parameters. This behavior was because the response of the HC-FCS columns under vehicle impact was slower than the RC columns because of their lower stiffness (see Figure 4). As the PTMSA is a moving average, gradual change in the dynamic impact force with time before and after the PDF leads to higher PTMSA. Hence, the PTMSA was higher for the case of the HC-FCS column than the RC column. This result was compatible with design Equations (1)–(4) by Abdelkarim and ElGawady [3,21] as Equation (3) or Equation (4) for the HC-FCS column reveals higher design force than that of the RC column using Equation (1) or Equation (2).

For the investigated values of all parameters other than vehicle velocity and mass, the static forces (PTMSAs) were lower than the static force from the AASHTO-LRFD of 2670 kN (600 kips) which was the normalized force of 1.0 in Figure 5b. However, when the vehicle velocity was 112 kph (70 mph) (Columns C17-R and C17-H), and the vehicle mass was 16 tons (30 kips) or higher (Columns C18-R, C18-H, C19-R, and C19-H), the static forces were higher than the static force from the AASHTO-LRFD. Therefore, the AASHTO-LRFD was found to be nonconservative for these six columns. The lower kinetic energy of these six columns was for the columns C17-R and C17-H which was 3870 kN.m (2855 kip.ft). So, the AAHSTO-LRFD was found to be nonconservative for the investigated columns when the kinetic energy was 3870 kN.m (2855 kip.ft) or higher.

### 4.2. Effects of the Parameters

**Figure 6:**
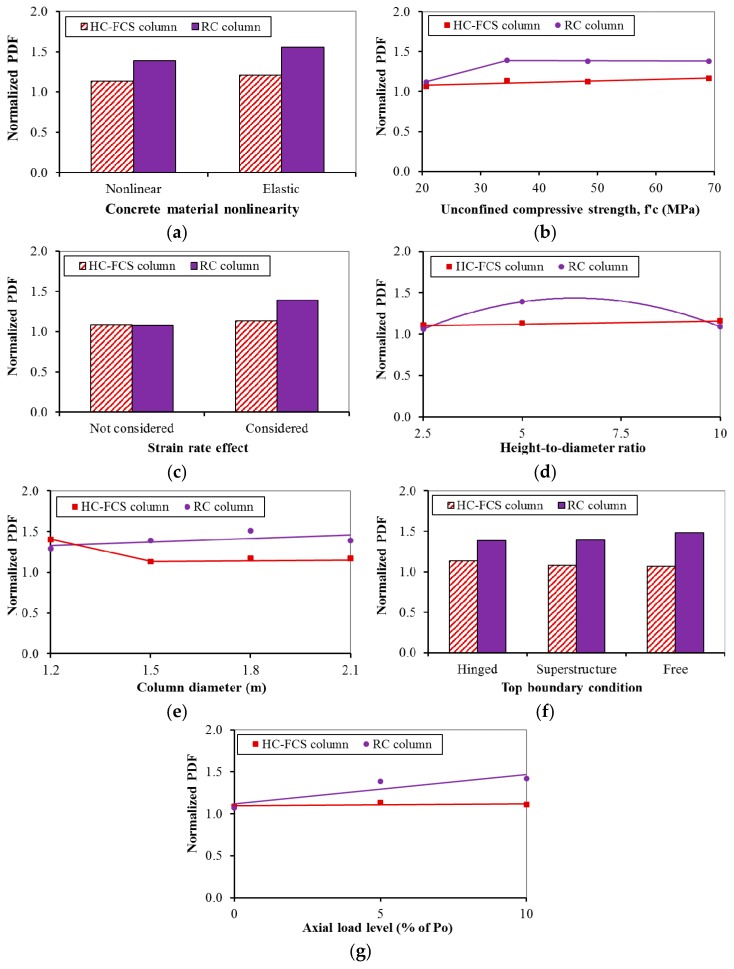
Effects of: (**a**) concrete material model; (**b**) concrete strength ($f_{c}^{\prime }$); (**c**) strain rate; (**d**) height-to-diameter ratio; (**e**) column diameter; (**f**) top boundary condition; and (**g**) axial load level on the PDF of the HC-FCS and RC columns.

**Figure 7:**
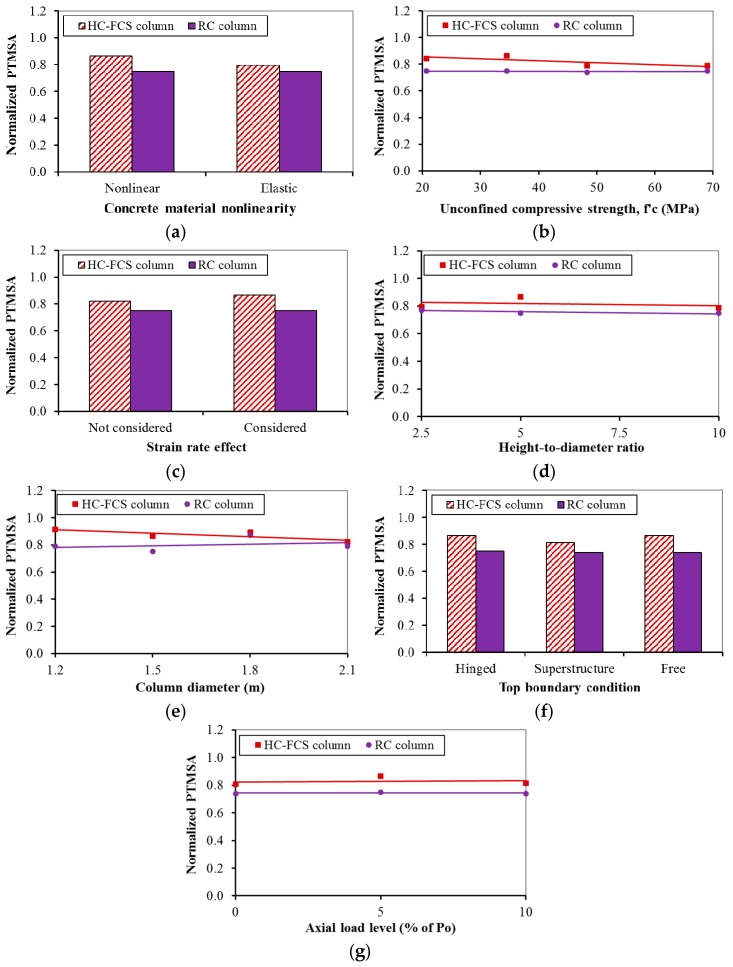
Effects of: (**a**) concrete material model; (**b**) concrete strength ($f_{c}^{\prime }$); (**c**) strain rate; (**d**) height-to-diameter ratio; (**e**) column diameter; (**f**) top boundary condition; and (**g**) axial load level on the PTMSA of the HC-FCS and RC columns.

**Figure 8:**
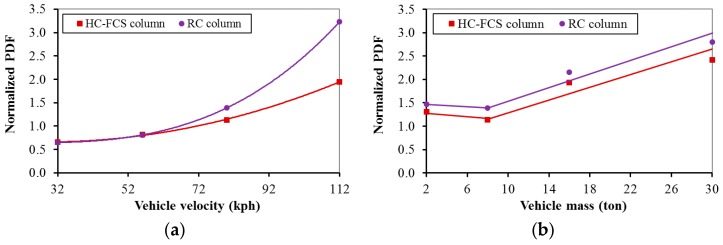
Effects of: (**a**) vehicle velocity and (**b**) vehicle mass on the PDF of the HC-FCS and RC columns.

**Figure 9:**
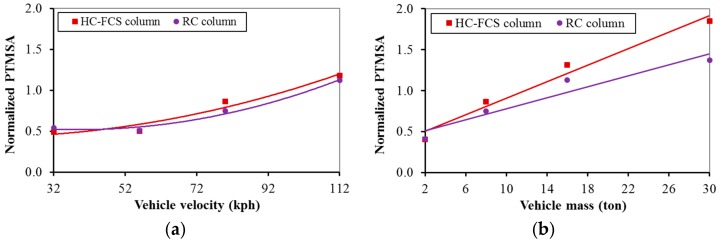
Effects of: (**a**) vehicle velocity and (**b**) vehicle mass on the PTMSA of the HC-FCS and RC columns.

Figure 6, Figure 7, Figure 8 and Figure 9 illustrate the comparisons between the HC-FCS and RC columns in terms of the PDF and the PTMSA through the investigated nine parameters. 

#### 4.2.1. Concrete Material Model

Using nonlinear concrete material for the columns led to a lower PDF than when using elastic material (Figure 6a and Figure 7a). This behavior occurred because the columns with nonlinear material had a higher deformation than those with linear material, leading to higher energy dissipation and hence reduction in the dynamic forces. In the other direction, the PTMSA as the maximum moving average calculated from the time-dynamic impact force relation had lower values when the curve was steep before and after the time of the PDF. Hence, the RC column had lower PTMSA than the HC-FCS column as it had a steeper curve near the PDF, because of its higher stiffness, than that of the HC-FCS column (see Figure 4). However, considering the nonlinear material affected the dynamic and static forces, the bridge designer may use the elastic material for simplicity as the effect of the nonlinearity on the static forces was lower than 10%.

#### 4.2.2. Concrete Strength ($f_{c}^{\prime }$)

In general, when the $f_{c}^{\prime }$ increased, the PDF and the PTMSA did not change in the case of the RC columns (Figure 6b and Figure 7b). The PDF and PTMSA slightly increased and decreased, respectively, in the case of the HC-FCS columns (Figure 6b and Figure 7b). The reason that the $f_{c}^{\prime }$ effect was not significant was because the vehicle impact is mainly dominated by shear forces as the applied impact load is usually close to the footing, and the main resistance for shear comes from the reinforcements. It was worth noting that when the $f_{c}^{\prime }$ was 20.7 MPa (3000 psi), the PDF of the RC and the HC-FCS columns were almost the same. This behavior was because of the high-energy dissipation in the form of concrete cover spalling in the case of the RC column which recovered the effect of the difference in the stiffness between the two columns. Also, the energy dissipation due to the concrete cover spalling was the reason for the lower PDF of the RC column with the $f_{c}^{\prime }$ of 20.7 MPa (3000 psi) than that of the other columns with higher $f_{c}^{\prime }$. The bridge designer may consider no changes in the behavior of the RC or the HC-FCS columns under vehicle collision with changing the $f_{c}^{\prime }$. However, an $f_{c}^{\prime }$ of 34.5 MPa (5000 psi) or higher is recommended for the RC column to avoid high concrete cover spalling.

#### 4.2.3. Materials Strain Rate

The PDF of the RC column increased when considering the strain rate, although the PTMSA was not affected (Figure 6c and Figure 7c). However, the PDF and PTMSA increased slightly when considering the strain rate for the HC-FCS column (Figure 6c and Figure 7c). In general, the strain rate of the vehicle impact is relatively low [35]. Therefore, the steel and FRP properties did not increase noticeably because of this low strain rate. However, the strain rate considerably increased the concrete tensile strength (Equations (12)–(15)) and hence reduced the concrete tension cracks. Therefore, the dynamic force of the RC column decreased by excluding the strain rate effect as the concrete damage increased leading to higher energy dissipation. In the other direction, the concrete shell of the HC-FCS column was confined by the FRP tube which already increased the concrete strength and protected it from spalling during the collision. Therefore, there was no significant difference in the results when the strain rate effect was excluded in the case of the HC-FCS columns. As the PTMSAs of both columns were not affected by considering the strain rate, the bridge designer may neglect the effect of the strain rate and use the designated material properties.

#### 4.2.4. Columns’ Height-to-Diameter Ratio

Changing the height-to-diameter (*H/D_o_*) ratio had a negligible effect on the PDF of the HC-FCS columns and the PTMSA of the RC and HC-FCS columns (Figure 6d and Figure 7d). This behavior was because the vehicle impact point of application is close to the footing of the columns. Therefore, vehicle collisions with bridge columns are usually dominated by shear force, which is controlled mainly by the cross-section configuration. The PDF of the RC columns behaved nonlinearly with changing the *H/D_o_* where the PDF increased when the *H/D_o_* increased until a ratio of approximately 6, where it then decreased. The reason was the concrete cover spalling with the small height and *H/D_o_* of 2.5 was considerably high as a percent of the height leading to high energy dissipation and hence low PDF. In the other direction, the concrete cover spalling with a long height and *H/D_o_* of 10 was considerably low as a percent of the height, but the longitudinal reinforcement buckling was high, leading to high energy dissipation and a consequently low PDF. However, these types of damages in the case of the RC columns did not exist in the case of the HC-FCS columns. Therefore, the behavior of the PDF of the RC column was different than that of the HC-FCS column.

#### 4.2.5. Columns’ Diameter

In general, the PDF and PTMSA increased slightly when the RC column diameter increased because the shear strength of the column increased (Figure 6e and Figure 7e). However, the shear strength of the HC-FCS column increased with an increase in diameter as the steel tube diameter and thickness grew with constant *D_i_/t_s_* of 75, but the PDF of the columns with diameters ≥1500 mm (5.0 ft) was lower than that of the column with a diameter of 1200 mm (4.0 ft) (Figure 6e). This behavior was because the local deformation of the steel tubes of the columns with diameters ≥1500 mm (5.0 ft) was considerably higher than that of the column with a diameter of 1200 mm (4.0 ft) due to the difference of the tube curvature in cross-section. Abdelkarim and ElGawady [21] stated that the main resistance of the HC-FCS columns under vehicle collision is from the inner steel tube and related the resistance to the tube curvature. The PTMSA of the HC-FCS columns decreased slightly when the diameter increased (Figure 7e).

#### 4.2.6. Columns’ Top Boundary Conditions

The PDF and PTMSA were almost constant when changing the top boundary condition for the both types of columns (Figure 6f and Figure 7f). The reason for this behavior was the very short duration of the impact event comparable to the natural period of the columns. Therefore, the columns’ response was mainly controlled by the amplitude of the imposed kinetic energy by the vehicle. Similar behavior was explained for pulse loads by Chopra [36]. Therefore, the bridge designer does not need to simulate the bridge deck to investigate the behavior of the bridge column under vehicle impact and could consider the top boundary condition as free or hinge-based on the most applicable status for the bridge being studied.

#### 4.2.7. Axial Load Level

The PDF of the RC column increased when the axial load increased as the axial compressive stresses imposed on the concrete elements delayed the tension cracks developed by the vehicle collision and hence reduced energy dissipation (Figure 6g and Figure 7g). The PDF of the HC-FCS columns was not affected by the axial compressive stresses on the concrete shell as the main resistance of such columns under vehicle collision comes from the steel tube only [21]. The PTMSAs were almost constant for both types of columns. Therefore, the bridge designer does not need to consider the applied axial compressive loads on bridge columns during vehicle collision if the axial load level is ≤ 10% of the axial capacity.

#### 4.2.8. Vehicle Characteristics (Mass and Velocity)

The PDF and the PTMSA increased nonlinearly with the increase of the vehicle velocity for the both types of columns (Figure 8a and Figure 9a). In general, the PDF and the PTMSA increased linearly with the increase of the vehicle mass for both of the columns (Figure 8b and Figure 9b). When changing the vehicles’ characteristics (mass and velocity), the behavior of the columns was compatible with the applied kinetic energy of the colliding truck as the kinetic energy is linear with the mass and nonlinear with the velocity where the kinetic energy is half of the vehicle mass times the square of the vehicle velocity (KE = ½ mv^2^). Therefore, the vehicle velocity and mass were the parameters that most affected the dynamic and static forces for both types of columns.

## 5. Summary and Conclusions

The behavior of HC-FCS and RC columns under vehicle collision was compared through nine parameters including the concrete material model, unconfined concrete compressive strength, material strain rate, column height-to-diameter ratio, column diameter, column top boundary condition, axial load level, vehicle velocity, and vehicle mass. The HC-FCS column consisted of an outer FRP tube, an inner steel tube, and a concrete shell sandwiched between the two. The steel tube was hollow inside and embedded into the concrete footing at a depth of 1.5 times the tube diameter while the FRP tube stopped at the top of the footing. The RC column had a solid cross-section. The following conclusions were revealed:

1. It was found generally that the HC-FCS columns had lower dynamic forces and higher static forces than the RC columns when changing the values of the different parameters.
2. The dynamic and static forces of the vehicle’s collision with either the RC or the HC-FCS columns were affected mainly by the vehicle’s velocity and mass.
3. For simplicity, bridge designers could consider linear behavior of the concrete and exclude the strain rate effect when designing columns under vehicle collision. Also, the $f_{c}^{\prime }$ of 34.5 MPa (5000 psi) or higher is recommended for the RC column to avoid high concrete cover spalling.
4. The bridge designer does not need to simulate the bridge deck to investigate the behavior of the bridge column, and the axial load can be excluded if it is ≤10% of the column’s axial capacity.

## Figures and Tables

**Table 1:** Summary of the examined columns’ parameters.

RC Column	HC-FCS Column	Concrete Material	$f_{c}^{\prime }$, MPa (Psi)	Strain Rate (*SR*)	Height-to-Diameter, (*H*/*D_o_*)	Outer Diameter (*D_o_*), m (ft)	Top Boundary Condition	Axial Load Level of *P_o_*	Vehicle Velocity (*v_r_*), Kph (Mph)	Vehicle Mass (*m*), Ton (Kip)
C0-R	C0-H	Nonlinear	34.5 (5000)	Considered	5	1.5 (5.0)	Hinged	5%	80 (50)	8 (18)
C1-R	C1-H	Elastic	34.5 (5000)	Considered	5	1.5 (5.0)	Hinged	5%	80 (50)	8 (18)
C2-R	C2-H	Nonlinear	20.7 (3000)	Considered	5	1.5 (5.0)	Hinged	5%	80 (50)	8 (18)
C3-R	C3-H	Nonlinear	48.3 (7000)	Considered	5	1.5 (5.0)	Hinged	5%	80 (50)	8 (18)
C4-R	C4-H	Nonlinear	69.0 (10,000)	Considered	5	1.5 (5.0)	Hinged	5%	80 (50)	8 (18)
C5-R	C5-H	Nonlinear	34.5 (5000)	Not considered	5	1.5 (5.0)	Hinged	5%	80 (50)	8 (18)
C6-R	C6-H	Nonlinear	34.5 (5000)	Considered	2.5	1.5 (5.0)	Hinged	5%	80 (50)	8 (18)
C7-R	C7-H	Nonlinear	34.5 (5000)	Considered	10	1.5 (5.0)	Hinged	5%	80 (50)	8 (18)
C8-R	C8-H	Nonlinear	34.5 (5000)	Considered	5	1.2 (4.0)	Hinged	5%	80 (50)	8 (18)
C9-R	C9-H	Nonlinear	34.5 (5000)	Considered	5	1.8 (6.0)	Hinged	5%	80 (50)	8 (18)
C10-R	C10-H	Nonlinear	34.5 (5000)	Considered	5	2.1 (7.0)	Hinged	5%	80 (50)	8 (18)
C11-R	C11-H	Nonlinear	34.5 (5000)	Considered	5	1.5 (5.0)	Free	5%	80 (50)	8 (18)
C12-R	C12-H	Nonlinear	34.5 (5000)	Considered	5	1.5 (5.0)	Superstructure	5%	80 (50)	8 (18)
C13-R	C13-H	Nonlinear	34.5 (5000)	Considered	5	1.5 (5.0)	Hinged	0%	80 (50)	8 (18)
C14-R	C14-H	Nonlinear	34.5 (5000)	Considered	5	1.5 (5.0)	Hinged	10%	80 (50)	8 (18)
C15-R	C15-H	Nonlinear	34.5 (5000)	Considered	5	1.5 (5.0)	Hinged	5%	112 (70)	8 (18)
C16-R	C16-H	Nonlinear	34.5 (5000)	Considered	5	1.5 (5.0)	Hinged	5%	56 (35)	8 (18)
C17-R	C17-H	Nonlinear	34.5 (5000)	Considered	5	1.5 (5.0)	Hinged	5%	32 (20)	8 (18)
C18-R	C18-H	Nonlinear	34.5 (5000)	Considered	5	1.5 (5.0)	Hinged	5%	80 (50)	2 (4.4)
C19-R	C19-H	Nonlinear	34.5 (5000)	Considered	5	1.5 (5.0)	Hinged	5%	80 (50)	16 (35)
C20-R	C20-H	Nonlinear	34.5 (5000)	Considered	5	1.5 (5.0)	Hinged	5%	80 (50)	30 (65)
